# The correlation between dysfunctional intestinal flora and pathology feature of patients with pulmonary tuberculosis

**DOI:** 10.3389/fcimb.2022.1090889

**Published:** 2022-12-21

**Authors:** Shiqing Ye, Liang Wang, Shengkai Li, Qingyong Ding, Yu Wang, Xinxin Wan, Xiaoyun Ji, Yongliang Lou, Xiang Li

**Affiliations:** ^1^ Wenzhou Key Laboratory of Sanitary Microbiology, Key Laboratory of Laboratory Medicine, Ministry of Education, China, Zhejiang Provincial Key Laboratory of Medical Genetics, School of Laboratory Medicine and Life Sciences, Wenzhou Medical University, Wenzhou, Zhejiang, China; ^2^ Colorectal Cancer Research Center, Wenzhou Medical University, Wenzhou, Zhejiang, China

**Keywords:** pulmonary tuberculosis, 16S rRNA gene sequencing, gut microbiota, biochemical index, SCFA

## Abstract

**Introduction:**

Recent studies have provided insights into the important contribution of gut microbiota in the development of Pulmonary Tuberculosis (PTB). As a chronic consumptive infectious disease, PTB involves many pathological characteristics. At present, research on intestinal flora and clinical pathological Index of PTB is still rare.

**Methods:**

We performed a cross-sectional study in 63 healthy controls (HCs) and 69 patients with untreated active PTB to assess the differences in their microbiota in feces via 16S rRNA gene sequencing.

**Results:**

Significant alteration of microbial taxonomic and functional capacity was observed in PTB as compared to the HCs. The results showed that the alpha diversity indexes of the PTB patients were lower than the HCs (P<0.05). Beta diversity showed differences between the two groups (P<0.05). At the genus level, the relative abundance of Bacteroides, Parabacteroides and Veillonella increased, while Faecalibacterium, Bifidobacterium, Agathobacter and CAG-352 decreased significantly in the PTB group, when compared with the HCs. The six combined genera, including Lactobacillus, Faecalibacterium, Roseburia, Dorea, Monnoglobus and [Eubacterium]_ventriosum_group might be a set of diagnostic biomarkers for PTB (AUC=0.90). Besides, the predicted bacterial functional pathway had a significant difference between the two groups (P<0.05), which was mainly related to the nutrient metabolism pathway. Significant alterations in the biochemical index were associated with changes in the relative abundance of specific bacteria, the short chain fatty acid (SCFA)-producing bacteria enriched in HCs had a positively correlated with most of the biochemical indexes.

**Discussion:**

Our study indicated that the gut microbiota in PTB patients was significantly different from HCs as characterized by the composition and metabolic pathway, which related to the change of biochemical indexes in the PTB group. It was hypothesized that the abovementioned changes in the gut microbiota could exert an impact on the clinical characteristics of PTB through the regulation of the nutrient utilization pathway of the host by way of the gut-lung axis.

## Introduction

A large number of microorganisms exist in the human body, especially the intestinal microbiome, which plays a crucial role in human physiology and pathology (de Vos et al., 2022). The gut microbiota will not only directly modulate the intestinal tract but also impact the distal organs, such as the brain, liver and lung (Zhu et al., 2021). Recently, an increasing amount of evidence has indicated that the gut microbiota is closely related to respiratory diseases, such as asthma, chronic obstructive pulmonary disease (COPD), lung cancer and respiratory infection (Dumas et al., 2018; Barcik et al., 2020; Li et al., 2021; Zhao et al., 2021). Pulmonary tuberculosis (PTB) is one of the most common infectious diseases and remains a major infectious cause of death worldwide (Pezzella, 2019). Emerging scientific research hints at a possible role of the gut microbiota in the pathophysiology of tuberculosis (TB) (Comberiati et al., 2021).

A study performed in South Africa depicted the distinct stool microbiome in the cases of tuberculosis with features of enriched anaerobes, which related to the upregulation of pro-inflammatory immunological pathways (Naidoo et al., 2021). Another study reported in India showed different results, researchers observed that butyrate and propionate-producing bacteria were significantly enriched in TB patients (Maji et al., 2018). As a country with a high burden of tuberculosis, researches on tuberculosis and flora have been widely implemented in China. In the east of China, compared with the healthy controls, the gut microbiota in the TB group was reflected by a striking decrease in short-chain fatty acids (SCFAs)-producing bacteria as well as metabolically associated pathways (Hu et al., 2019). Importantly, a study performed in the west of China showed a positive correlation between the gut microbiota and peripheral CD4^+^ T cell counts in the TB patients (Luo et al., 2017), which highlighted the associations between gut microbiota with tuberculosis and its clinical outcomes. Although many studies described that the change of bacterial function is related to PTB (Wang et al., 2022; Yang et al., 2022), the specific mechanism between flora and clinical characteristics remains unclear.

Herein, we have performed a comparative analysis of 63 healthy controls (HCs) and 69 PTB patients based on 16S rRNA sequencing. We found that the intestinal flora of the PTB group was characterized by the increase of proinflammatory bacteria and the decrease of SCFAs-producing bacteria, which was related to the metabolic pathway of nutrients. Notably, we set a series of specific genera as novel biomarkers for identification between PTB patients and HCs. More importantly, we found that some probiotics enriched in HCs were positively correlated with most of the biochemical indexes, indicating that the enrichment of probiotics in the intestine was closely related to the healthy physiological state of the host. In general, we aim to identify the association with the microbiome, biochemical index and pulmonary tuberculosis.

## Material and methods

### Ethics

This study was approved by the Ethics Review Committee of Affiliated Dongyang Hospital of Wenzhou Medical University (2022-YX-268), and written informed consent was obtained from all participants.

### Participant recruitment

A total of 69 pulmonary tuberculosis patients and 63 healthy controls were initially enrolled from the Affiliated Dongyang Hospital of Wenzhou Medical University, Zhejiang, China, from May 2021 to June 2022. All patients were recruited on the basis of TB symptomatic features (WS288-2008). Healthy controls (HCs) were recruited in hospital health examination center, who did not have close contact with TB patients at least one year. All participants had not taken any broad-spectrum antibiotics in the previous six months and without other basic diseases or major illness.

### Biochemical analysis

Blood samples were collected for biochemical analysis using the automated equipment and standard methods. The level of Albumin (Alb), Serum fasting blood glucose (Glu), Total Cholesterol (TC), Triglycerides (TG), High-Density Lipoprotein cholesterol (HDL), Low-Density Lipoprotein cholesterol (LDL), and Creatinine (Cr) were examined using a Hitachi 7600 automatic biochemical analyzer (HITACHI, Japan). The Hemoglobin (Hb) and White Blood Cell (WBC) were examined by Sysmex pocH-100i automatic blood Analyzer (SYSMEX, Japan).

### Fecal sample collection and microbe DNA extraction

Fresh stool samples (morning samples, 200 mg/aliquot) were collected using GUHE Flora Storage buffer (GUHE Laboratories, Hangzhou, China), and stored at room temperature until DNA extraction. DNA was extracted with nucleic acid extraction or purification reagent (GUHE Laboratories, GHFDE100, Hangzhou, China), and the quantity and quality of extracted DNAs were measured using the NanoDrop ND-1000 spectrophotometer (Thermo Fisher Scientific, Waltham, MA, USA) and agarose gel electrophoresis, respectively.

### 16S rRNA gene sequencing

The V4 hypervariable regions of the bacterial 16S rRNA gene were amplified from each sample using the 515F(5’-GTGCCAGCMGCCGCGGTAA-3’) and 806R(5’-GGACTACHVGGGTWTCTAAT-3’) primers as previously described prior to sequencing. Phusion High-Fidelity PCR Master Mix with HF Buffer (PHUSION high fidelity DNA polymerase) was used for PCR. The following primary PCR cycling conditions were used: (1) Pre-denaturatio at 98℃ for 30 s; (2) 25 cycles of PCR at 98℃ for 15 s, 58℃ for 15 s, and 72℃ for 15 s; (3) a final extension at 72℃ for 1 min. PCR products were purified with AMPure XP Beads (Beckman Coulter, Indianapolis, IN), and the library was quantified with Qubit. After quantification, the Illumina NovaSeq6000 platform at GUHE Info Technology (GUHE, Hangzhou, China) was used for sequencing. The sequence data of this study have been uploaded in the GenBank Sequence Read Archive (SRA) of NCBI under the accession code BioProject PRJNA901399.

### Sequence analysis

The original data of each sample were split according to the Barcode sequence and the primer sequence. The low-quality sequences were filtered through following criteria (Gill et al., 2006; Chen and Jiang, 2014): sequences that had a length of <150 bp, sequences that had average Phred scores of <20, sequences that contained ambiguous bases, and sequences that contained mononucleotide repeats of >8 bp. Paired-end reads were assembled using Vsearch V2.4.4 (–fastq_mergepairs –fastq_minovlen 5), than Operational taxonomic unit (OTU) picking using Vsearch v2.15.0, included dereplication(–derep_fulllength), cluster(-cluster_unoise), detectection of chimeras(-uchime3_denovo) (Rognes et al., 2016). The final effective tags were obtained by removing the chimeric sequences.

### OTU/ASV clustering and species annotation

The filtered clean reads were clustered to get ASV statistical results. The representative sequences of OTUs were selected using default parameters. The representative sequences were annotated through QIIME2 (v2020.6) based on the SILVA138 database to further generate the OTU list. At each classification level, phylum, family, and genus were used to count the community composition of each sample.

### Bioinformatics and statistical analysis

Sequence reads for 16S rRNA gene amplicons were analyzed using QIIME2 software and R package (v3.2.0). QIIME2 was used to calculate alpha diversity index in OTU level, including Chao1, ACE, Shannon, Simpson index. Beta diversity analysis was calculated through Qimme software based on UniFrac distance metric (Lozupone and Knight, 2005; Lozupone et al., 2007). “Vegan” from R package was used to evaluate the markers of microbial community structure differentiation between groups through PERMANOVA (Permanent multivariate analysis of variance) (McArdle and Anderson, 2001). The classification and abundance of bacteria were visualized by MEGAN software and GraPhlAn. And the R package “VennDiagram” was used to generate a Venn diagram (Zaura et al., 2009). Taxa abundances at the phylum, class, order, family, genus and species levels were statistically compared among samples or groups by Kruskal test from R stats package (metagenomeSeq packages). LEfSe (Linear discriminant analysis effect size) was performed to detect differentially abundant taxa across groups using the default parameters (Segata et al., 2011). Microbial functions were predicted by PICRUSt2 (Phylogenetic investigation of communities by reconstruction of unobserved states, https://github.com/picrust/picrust2/), based on high-quality sequences. For each sample, functions were profiling by using the Omixer-RPM version 1.0 (https://github.com/raeslab/omixer-rpm) with KO redundants predicted from PICRUSt2. Spearman’s rank correlation between the intestinal flora and biochemical indexes was calculated with our own python scripts (Ramsey, 1989). Random forest analysis was applied to discriminating the samples from different groups using the R package “random Forest” with 1,000 trees and all default settings. Values were expressed as the mean ± SEM. All other comparisons were calculated by two-tailed Student’s t-test or one-way ANOVA followed by Tukey’s test using GraphPad Prism 6 software.

## Results

### Participant cohort

**Table 1** showed the detailed clinical characteristics of the study participants. 69 PTB patients and 63 age- and sex-matched healthy controls (HCs) were enrolled in this study. Among the clinical biochemical indexes tested in the study, a significant increase in serum fasting blood glucose (Glu) was observed in PTB patients (*P*<0.05). The levels of Albumin (Alb), Creatinine (Cr), Hemoglobin (Hb), Triglycerides (TG), Total cholesterol (TC), High-density lipoprotein cholesterol (HDL) and Low-density lipoprotein cholesterol (LDL), were all found to be significantly lower in PTB patients than in HCs (*P*<0.001). There was no statistical difference in White blood cells (WBC) between the two groups.

**Table 1:** Clinical characteristics of the participants.

Variable	HC (n=63) mean (range)	PTB (n=69) mean (range)	Student’s t test (*P* values)
Gender, Man, n (%)	40 (63.49%)	44 (63.77%)	n.s.
Age (year)	49 (34-67)	51 (30-69)	n.s.
Albumin (g/L)	46.86 (41.60-51.80)	32.62 (14.90-44.60)	< 0.001
Creatinine (mmol/L)	75 (48-105)	63(35-110)	< 0.001
Hemoglobin (g/L)	150 (110-170)	121 (70-175)	< 0.001
Triglycerides (mmol/L)	1.64 (0.54-5.04)	1.03(0.31-2.71)	< 0.001
Total cholesterol (mmol/L)	4.34 (2.33-6.35)	3.34(1.36-5.99)	< 0.001
High-density lipoprotein (mmol/L)	1.26(0.79-1.98)	0.96(0.12-1.88)	< 0.001
Low-density lipoprotein (mmol/L)	3.03 (0.95-5.31)	2.13(0.46-3.89)	< 0.001
Serum fasting blood glucose (mmol/L)	5.08 (4.22-7.04)	6.19(4.27-21.29)	< 0.05
White blood cell (×10^9^/L)	6.13(3.46-9.10)	6.36(3.07-13.55)	n.s.

HC, healthy control; PTB, pulmonary tuberculosis; n.s., non-significance difference.

### Dysbiosis of gut microbiota in PTB patients

**Figure 1:**
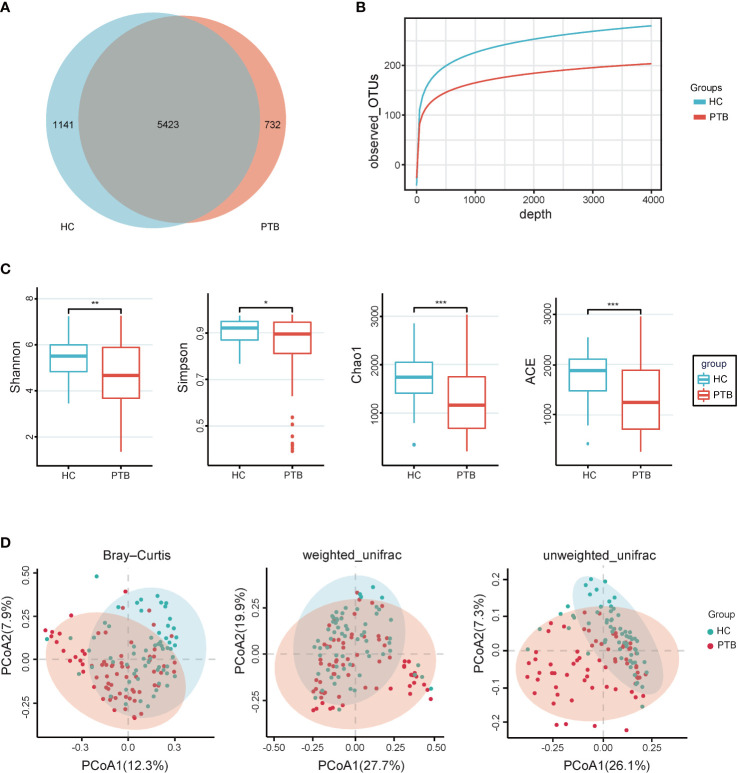
Intestinal microbial diversity. **(A)** Venn diagram showing the shared and unique OTUs in the gut microbiota of the two groups. **(B)** The rarefaction curves of two groups tended to be flat, indicating that the amount of sequencing data is large enough to reflect the majority of microbial species information in the sample. **(C)** The alpha diversity was assessed using the ACE, Chao1, Shannon and Simpson indexes, which showed significant differences between the PTB and HCs groups. *P<0.05, **P<0.01, ***P<0.001. Wilcoxon rank-sum test followed by Benjamini–Hochberg false discovery rate (FDR) correction. **(D)** PCoA based on Bray-Curtis, weighted_unifrac and Unweighted_Unifrac distance showed that gut microbiota in PTB separated from HCs (*P*<0.05). The significance was assessed by permutational multivariate analysis of variance (PERMANOVA) using the R package “vegan”. PTB, pulmonary tuberculosis; HCs, healthy controls.

In total, we generated 16,259,048 high-quality reads, and the reads were clustered into 7296 operational taxonomic units (OTUs) based on a similarity of 97%. A Venn diagram showed that 1141 OTUs and 732 OTUs were unique in the healthy controls and PTB patients, respectively (**Figure 1A**). The rarefaction curves showed that the curve entered in plateau, indicating that the sequencing data was large enough to reflect the bacterial diversity of the intestinal flora in HCs was higher than that in the PTB patients (**Figure 1B**). The alpha diversity indexes, including Shannon, Simpson, Chao1 and ACE reflected that the diversity of fecal microbiota in PTB patients was significantly lower than in HCs (*P*<0.01, *P*<0.05, *P*<0.001, *P*<0.001, respectively) (**Figure 1C**). The principal coordinate analysis (PCoA), based on Bray-Curtis, unweighted UniFrac and weighted UniFrac distances were performed to identify the difference in fecal microbiota composition between PTB patients and HCs. As shown in **Figure 1D**, there was a tendency of separation and had a partial overlap between two groups, which demonstrated that the total community of gut microbiota in the two groups was different.

**Figure 2:**
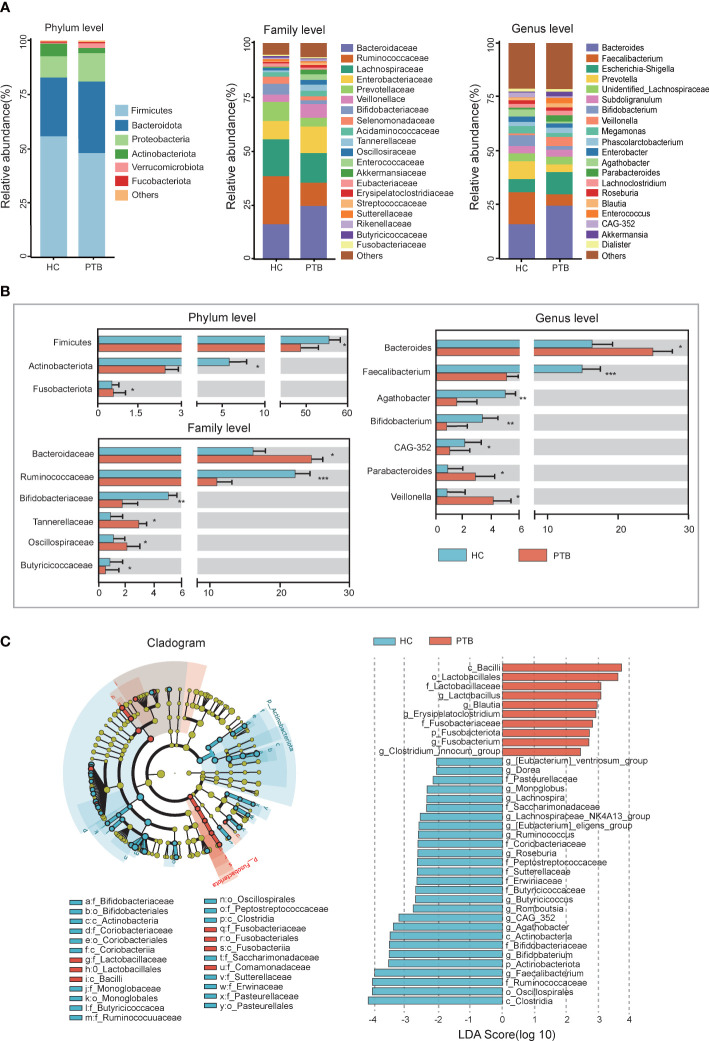
Relative abundance and LEfSe analysis of intestinal microbiota. **(A)** The relative abundance of intestinal flora between the PTB patients (red) and HCs (blue) at the phylum level, the family level and the genus level. **(B)** The statistical difference of intestinal flora between two groups. *P<0.05, **P<0.01, ***P<0.001. The significance was assessed by Student’s t test. **(C)** The cladogram identified the differentially abundant taxa between the PTB patients and HCs. The HC-enriched taxa were indicated with a negative LDA score (blue), and the taxa enriched in the patients with PTB were characterized by a positive score (red).

The compositions of the fecal microbiota in the PTB patients and the HCs were assessed at different taxonomic levels, and the top 20 relative abundance of bacteria were shown in **Figure 2A**. At the phylum level, we observed an enrichment of *Bacteroidota*(33.10% vs. 27.08%), *Proteobacteria*(12.99% vs. 9.83%) and *Fusobacteriota*(0.58% vs. 0.56%, *P*<0.05), and a remarkable decrease of *Firmicutes*(48.07% vs. 55.89%, *P*<0.05) and *Actinobacteriota*(2.44% vs. 5.83%, *P*<0.01) in PTB patients compared with HCs (**Figure 2B**). At the family level, the relative abundance of *Bacteroidaceae* (24.59% vs. 16.14%, *P*<0.05), *Tannerellaceae* (2.88% vs. 0.90%, *P*<0.05) and *Oscillospiraceae* (2.19% vs. 1.18%, *P*<0.05) increased significantly in PTB patients, while *Bifidobacteriaceae* (1.7% vs. 5.03%, *P*<0.01), *Butyricicoccaceae* (0.45% vs. 0.81%, *P*<0.05) and *Ruminococcaceae* (10.74% vs. 22.20%, *P*<0.001) decreased drastically (**Figure 2B**). At the genus level, compared with the HCs, the PTB patients had a significantly higher relative abundance of *Bacteroides* (25.59% vs. 16.14%, *P*<0.05), *Parabacteroides* (2.88% vs. 0.90%, *P*<0.05) and *Veillonella* (4.24% vs. 0.84%, *P*<0.05), and lower relative abundance of *Faecalibacterium* (5.31% vs. 14.80%, *P*<0.05), *Bifidobacterium* (1.67% vs. 5.02%, *P*<0.05), *Agathobacter* (0.86% vs. 3.32%, *P*<0.05) and *CAG-352* (0.60% vs. 2.21%, *P*<0.05) (**Figure 2B**). Our data indicated the altered gut microbiota composition in PTB patients.

LEfSe analysis demonstrated that many key taxa were clearly different between PTB patients and HCs. According to the LDA score, the significantly enriched taxa in the gut microbiome of the PTB patients were *Lactobacillus*, *Blautia*, *Erysipelatoclostridium* and *Fusobacterium*. While the abundance of *Faecalibacterium*, *Bifidobacterium*, *Butyricicoccus*, *Roseburia* and *Lachnospiraceae_NK4A136_group* were higher in HCs (**Figure 2C**). The structure of the intestinal flora was determined by dynamic interactions between these community members. Based on the relative abundance of OTUs, PTB patients showed a more complex network of interactions than the HCs, with more positive and negative correlations among the microbiota (**Figure S1**).

### The biochemical index was correlated with gut microbiota

**Figure 3:**
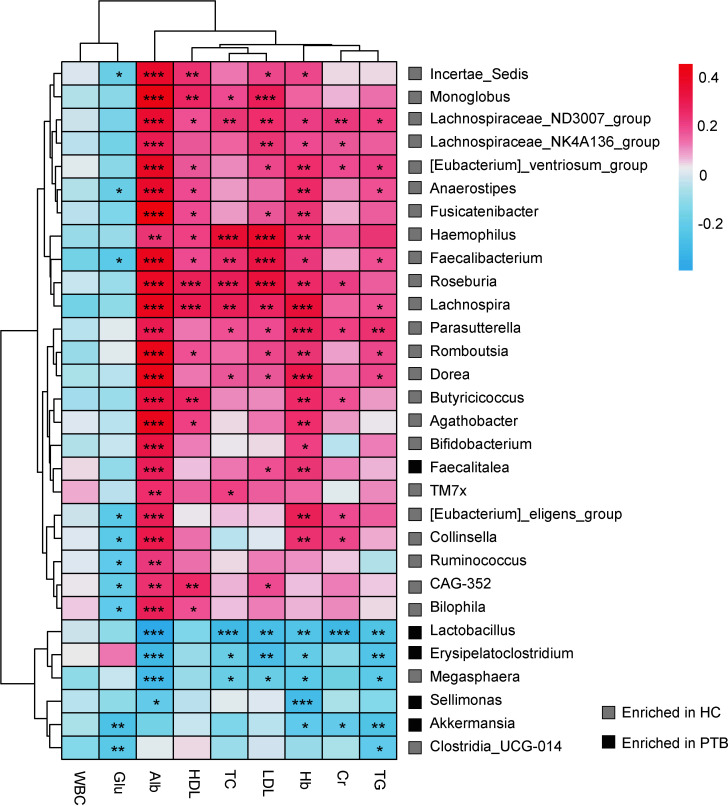
Correlation analysis between intestinal flora and biochemical indexes. The depth of the color in the heat maps signifies the strength of the correlation: red represents a positive correlation, whereas blue indicates a negative correlation. * *P*<0.05, ** *P*<0.01, *** *P*<0.001. The correlation were assessed by the Spearman test. Grey represents features enriched in HCs, while black represents features enriched in PTB patients.

A heat map was used to examine the associations between intestinal bacteria and the biochemical index (**Figure 3**). TC, HDL, LDL and Hb were positively correlated with 5 genera that were enriched in HCs, namely *Lachnospiraceae_ND3007_group*, *Haemophilus*, *Faecalibacterium*, *Roseburia*, *Lachnospira* (*P*<0.05). The levels of TG, Hb, LDL and TC were negatively associated with *Lactobacillus*, *Erysipelatoclostridium* and *Megasphaera* (*P*<0.05). Particularly, Alb was positively correlated with SCFA producers which were enriched in HCs, such as *Lachnospiraceae_NK4A136_group*, *Faecalibacterium*, *Dorea*, *Butyricicoccus* and *Bifidobacterium* (*P*<0.001), and negatively associated with *Lactobacillus*, *Erysipelatoclostridium*, *Megasphaera* and *Sellimonas*, most of which were enriched in PTB patients. Moreover, Cr was positively correlated with some bacteria enriched in HCs, and negatively correlated with bacteria enriched in PTB group (*P*<0.05). Besides, WBC had no direct relation with the genera in our study. It is also unusual in that Glu only had a negative correlation with *Incertae_Sedis*, *Faecalibacterium*, *[Eubactrium]_eligens_group*, *Collinsella*, *Ruminococcus*, *CAG-352*, *Bilophila*, *Akkermanisia* and *Clostridia_UCG-014* (*P*<0.05). Taken together, there was an intimate relationship between the deferential genera of PTB patients and the biochemical index, indicating that the altered gut microbiota may be the key pathophysiology of PTB.

### Microbial functional dysbiosis in PTB patients

**Figure 4:**
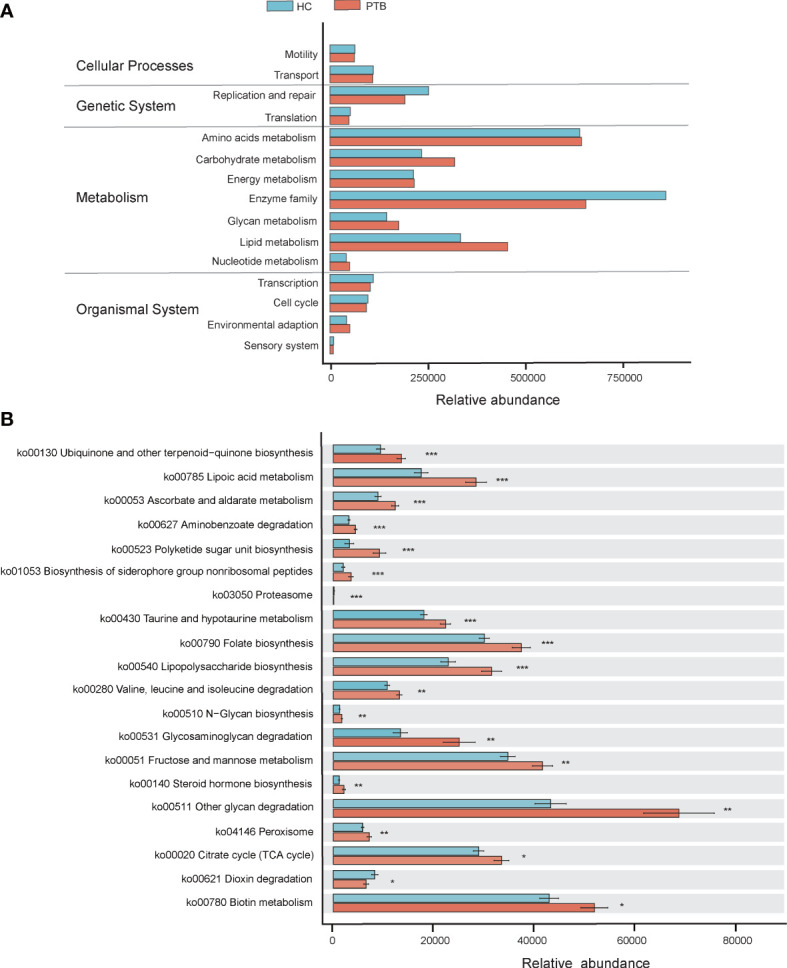
PICRUSt2-based examination of the fecal microbiome of the PTB patients and the healthy controls. **(A)** The different bacterial functions were evaluated between two groups. **(B)** A comparison of enriched KEGG pathways in PTB and HCs. * P<0.05, ** P<0.01, *** P<0.001. Functions and pathways were enriched in PTB group (red); Functions and pathways were enriched in the HC group (blue).

PICRUSt2 was used to identify the metabolic and functional changes in the fecal microbiota between the PTB patients and the HCs. The results of PICRUSt2 based on the KEGG classification showed that the predominant predicted bacterial functions were related to cellular processes, genetic system, metabolism and organismal system (**Figure 4A**). Top 20 pathways were shown in **Figure 4B**, among which 18 were overrepresented in the PTB patients, while only 2 were enriched in healthy controls. Compared with HCs, 7 biosynthesis pathways related to ubiquinone, polyketide, siderophore, folate, lipopolysaccharide, N-glycan and steroid hormone were highly represented in PTB patients. Interestingly, 5 metabolism pathways related to lipoic acid, ascorbate, taurine, fructose and biotin were strikingly increased in PTB patients. Moreover, the degradation capacities for different types of substrates, such as amino acid and glycan were increased in patients, while the dioxin degradation pathway was increased in HCs. Notably, PTB patients had more pathways involved in toxin degradation and energy circulation, specifically reflected in the rise of peroxisome and TCA cycle pathways. However, HCs obviously had more proteasome, indicating a superior protein utilization capacity (**Figure 4B**). Thus, these results further confirmed that *Mycobacterium tuberculosis* infection would disturb the functional of the fecal microbiota and might participate in the pathogenesis and development of PTB.

### Significant biomarkers based on gut microbiota for PTB diagnosis

**Figure 5:**
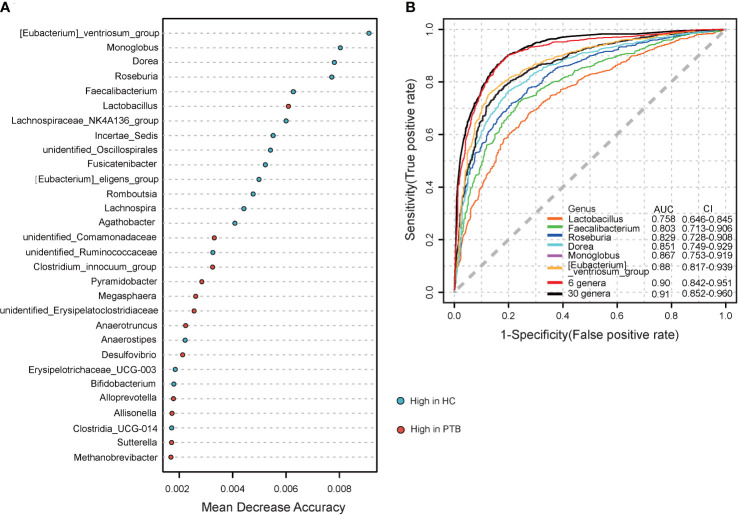
Establishing random forest models to predict the biomarkers of gut microbiota in PTBs. **(A)** The top 30 genera were selected to establish a random forest classifier for the diagnosis of PTB. The mean value enriched in HCs(blue); The mean value enriched in PTB patients (red). **(B)** Receiver operating characteristic (ROC) analysis of *Lactobacillu*, *Faecalibacterium, Roseburia, Dorea, Monnoglobus, [Eubacterium]_ventriosum_*group, 6 genera mentioned above and the 30 genera.

To further explore the diagnostic value of the gut microbiota for PTB, we constructed a random forest model to accurately distinguish PTB patients from HCs. 30 genus markers were selected from the model. The training of the random forest classifier involved the calculation of the feature importance of these 30 genera and their relative abundances (**Table S1**). The vital genera identified based on the model were described based on a mean decrease in accuracy (**Figure 5A**). ROC analysis was used to verify the diagnostic ability of these biomarkers. The area under the curve (AUC) obtained using the training datasets was 0.91, showing that patients with PTB could be successfully distinguished from the HCs. The AUC values for six genera, *Lactobacillus* (AUC=0.758), *Faecalibacterium* (AUC=0.803), *Roseburia* (AUC=0.829), *Dorea* (AUC=0.851), *Monnoglobus* (AUC=0.867) and *[Eubacterium]_ventriosum_group* (AUC=0.88), had an adequate diagnostic efficacy, respectively. Importantly, the model including all six genera had a relatively better diagnostic ability (AUC=0.90) (**Figure 5B**), indicating the combination of six genera may be a set of new diagnostic biomarkers for PTB.

## Discussion

Homeostatic interactions with the microbiome are central to healthy human physiology and nutrition is the main driving force shaping the microbiome (Dominguez-Bello et al., 2019; Gomaa, 2020). Malnutrition is one of the major factors in *Mycobacterium tuberculosis* (*Mtb*) infection, which has been associated with the gut microbiota (Martin and Sabina, 2019; Tellez-Navarrete et al., 2021). Once TB sets in, it leads to an increase in metabolism and a decrease in appetite that compounds the already present malnutrition (Hernández-Garduño and Pérez-Guzmán, 2007; Kant et al., 2015; Mashabela et al., 2019). Emerging studies have indicated cross-talk occurs between the gut microbiota and the pathogenesis of pulmonary tuberculosis (PTB) through a gut-lung axis (Dang and Marsland, 2019; Saint-Criq et al., 2021). Our study group consists of participants from eastern China, which is relatively less explored and has no consistent results in the existing research. We updated the data on the structure and function of the intestinal flora of Chinese tuberculosis patients and attempted to contact the interplay and mechanism between host nutritional status, pathology, and the gut microbiome.

We observed microbial dysbiosis in PTB patients, which was reflected in the reduction of diversity and the change of taxonomic composition (**Figures 1**, **2**), parallel to the recent study (Khaliq et al., 2021). In this study, at the phylum level, we found an enrichment of *Bacteroidota*, *Proteobacteria* and *Fusobacteriota* in PTB patients, many of which produced lipopolysaccharide (LPS), suggesting an increase of gut-derived LPS in PTB patients. Chronic infection and persistent inflammatory reaction of tuberculosis may affect the integrity of the intestinal mucosal barrier, facilitating the translocation of the bacteria, leading to the increase of circulating LPS, which further aggravated the inflammation of the host (Gallucci et al., 2021). Especially, as compared with the HCs, at the genus level, the abundance of *Bacteroides*, *Parabacteroides* and *Veillonella* increased markedly in PTB patients. The stability of gut microbiota is essential for the host’s health, while the enrichment of anaerobic bacteria, for example, *Bacteroides* and *Veillonella*, were associated with disease status, leading to inflammation (Wei et al., 2020; Zafar and Saier, 2021). Moreover, we observed a significant decrease in the abundance of SCFAs-producing genera in PTB patients, such as *Faecalibacterium*, *Bifidobacterium*, *Agathobacter* and *CAG-352* (**Figure 2A**). Especially, the depletion of *Faecalibacterium* and *Bifidobacterium* was related to the TB cases (Khaliq et al., 2021; He et al., 2021). It is known that SCFAs, especially, acetate, propionate and butyrate are key mediators of the beneficial effects elicited by the gut microbiome (Martin-Gallausiaux et al., 2021). In addition to maintain intestinal function, evidence is accumulating that SCFAs directly modulate host metabolic health related to appetite regulation, energy expenditure, glucose homeostasis and immunomodulation (Canfora et al., 2015; Blaak et al., 2020). More importantly, we found a set of novel non-invasive diagnostic biomarkers for PTB, the combination of 6 genera, containing *Lactobacillus*, *Faecalibacterium*, *Roseburia*, *Dorea*, *Monnoglobus* and *[Eubacterium]_ventriosum_group* specifically (**Figure 5**), had a relatively better diagnostic ability (AUC=0.90) than previous studies (Hu et al., 2019; Wang et al., 2022), which improved the accuracy and reduced the complexity.

Pulmonary tuberculosis (PTB) is a chronic consumptive disease, the malnutrition of PTB may cause anemia, hyperglycemia and other complications (Moon, 2014; Kant et al., 2015; Chhabra et al., 2021). In this study, we found that significantly lower lipid (TC, TG, HDL and LDL), Alb, Cr and Hb levels were other interesting characteristics of PTB patients (**Table 1**), which may result from a decrease in the utilization of protein and fat due to malnutrition (Kant et al., 2015). Moreover, we found that the levels of lipid, Alb and Hb were positively correlated with the abundances of SCFA-producing probiotics which enriched in HCs, such as *Lachnospiraceae_ND3007_group*, *Faecalibacterium*, *Roseburia*, *Dorea* and *Butyricicoccus* (**Figure 3**), indicating the enrichment of probiotics were related to the healthy physiological state of the host. *Faecalibacterium* and *Roseburia* are major producers of butyrate, which decreased *Mtb*-induced inflammation as well as insulin resistance (Jo et al., 2021; Faden, 2022). Meanwhile, the mentioned indexes were negatively correlated with the abundances of *Lactobacillus*, *Erysipelatoclostridium* and *Sellimonas*, which enriched in PTB patients (**Figure 3**). Previous study found that *Lactobacillus* was significantly increased in newly treated patients with PTB (Xie et al., 2021), and lactate, the product of *Lactobacillus*, was found to provide additional carbon matrix for *Mtb (* Billig et al., 2017). Besides, *Erysipelatoclostridium* was one of the major genera associated with active-TB, also associated with inflammation as well as lipid metabolism (Jo et al., 2021; Khaliq et al., 2021; Chang et al., 2022). In this study, we observed the level of Glu increased significantly in the PTB group (**Table 1**), and there was only a negative correlation between Glu level and the abundance of SCFAs producers, such as *[Eubacterium]_eligens_group*, *Ruminococcus* and *Akkermansia* (**Figure 3**) (Wang et al., 2022), different from the previous result (Hayashi et al., 2014). Although *Akkermansia* was reported to regulate blood glucose (Yoon et al., 2021), its ability might be reversed by other bacteria in the PTB group due to relative abundance or flora interaction. Thus, this study indicated that the imbalance of intestinal flora, especially the down-regulation of SCFA-producing bacteria, is related to the pathological indexes of PTB patients. In light of these observations, we considered this hypothesis that an upsurge of SCFAs-producing bacteria might have positive consequences on the host.

In addition to the SCFAs, we speculated another way that gut microbiota affected the pathological status of PTB patients was by regulating the bioavailability of nutrients such as amino acids, vitamins and glycan (Kant et al., 2015). The microbial function analysis revealed that PTB patients had a significant increase in the metabolism/degradation pathway of amino acids and glycan (**Figure 4B**), indicating an increased basal metabolic rate, which was consistent with the low fever pathology of PTB (El Amrani et al., 2016). The upregulation of these pathways probably resulted from the complicated microbial interaction network (**Figure S1**), which demanded much more nutrients to maintain in PTB patients than HCs. Notably, the lipopolysaccharide (LPS) biosynthesis pathway was significantly up-regulated in the PTB group, which was consistent with the up-regulated relative abundance of LPS-producing bacteria in PTB patients. Previous studies have found that LPS may be a sign of malnutrition and bacterial infection (Ubenauf et al., 2007; Patterson et al., 2021). Besides, LPS can induce insulin resistance, which is related to hyperglycemia (Salazar et al., 2020). The up-regulated LPS biosynthesis pathway may provide some explanations for the high blood glucose level and low blood lipid level of PTB patients in this study, which reaffirmed the involvement of gut microbiota in the process of PTB disease. Moreover, the biosynthesis of folic acid in this study increased significantly in PTB patients, it might be due to the enrichment of *Lactobacillus* in the PTB group, which was known as folic acid-producing bacteria (Levit et al., 2018; Hajian et al., 2019). Based on these results, the enrichment of proinflammatory bacteria in the PTB group seems to explain the pathologically elevated basal metabolic rate and the clinical characteristics of the host. Known as SCFAs can inhibit inflammation and regulate basic metabolism (Morrison and Preston, 2016; He et al., 2020), studies on the treatment of probiotics in diseases have been implemented (Xiong et al., 2021; Jiang et al., 2022). It seems feasible to treat PTB by replenishing the probiotics, for their excellent ability in improving the flora structure and increasing the production of SCFAs.

The results of this study provide a new framework for understanding the changes of intestinal microorganisms in the east China cohort exposed to *Mtb* and provide a new diagnostic marker for non-invasive diagnosis of PTB. We speculate that the enrichment of pro-inflammatory bacteria and the reduction of SCFA-producing probiotics in the PTB group caused the imbalance of intestinal flora structure and function, which led to the pathological metabolism in the PTB group, explaining the abnormality of clinical indexes in PTB patients. Therefore, it is meaningful to take probiotics to regulate the structure and function of the flora. The gut microbiota will be affected by environment, diet and living habits, diseases, antibiotics, etc. To increase the universality and strictness of this study, we need more large-scale follow-up research, including expanding cohort, upgrading sequencing methods, conducting animal experiments, etc., to obtain new insights from current findings. These studies will help us better understand the gut-lung axis and have potential significance for the non-invasive diagnosis and treatment of PTB.

## Data availability statement

The data presented in the study are deposited in the BioProject repository, accession number is PRJNA901399. The link for data can be found below: https://www.ncbi.nlm.nih.gov/bioproject/901399.

## Ethics statement

The studies involving human participants were reviewed and approved by Affiliated Dongyang Hospital of Wenzhou Medical University, Zhejiang, China. The patients/participants provided their written informed consent to participate in this study.

## Author contributions

Study concept and design: XL, YL; Specimen collection: LW,SY, QD; Analysis and interpretation of data and statistical analysis: SL, SY, XW, XJ; Drafting the manuscript: XL, SY, YW. The authors thank the GUHE Info technology, Co., Ltd., (Hangzhou, China) for expert technical advice. All authors contributed to the article and approved the submitted version.

## Supplementary material

Supplementary Figure 1 The interaction network of intestinal flora in HCs and PTB patients. The interaction network of intestinal flora in HCs showed a simple relationship between the community members, and the interaction network of intestinal flora in PTBs had more positive and negative correlations among the bacteria. The correlation coefficients were calculated with the Sparse Correlations for Compositional (SparCC) data algorithm. Cytoscape version 3.4.0 was used for network construction.
