# Supplementary figures and images for: The correlation between dysfunctional intestinal flora and pathology feature of patients with pulmonary tuberculosis
Source: Front Cell Infect Microbiol. 2022 Dec 21;12:1090889. doi: 10.3389/fcimb.2022.1090889 (PMC9811264; doi:10.3389/fcimb.2022.1090889)

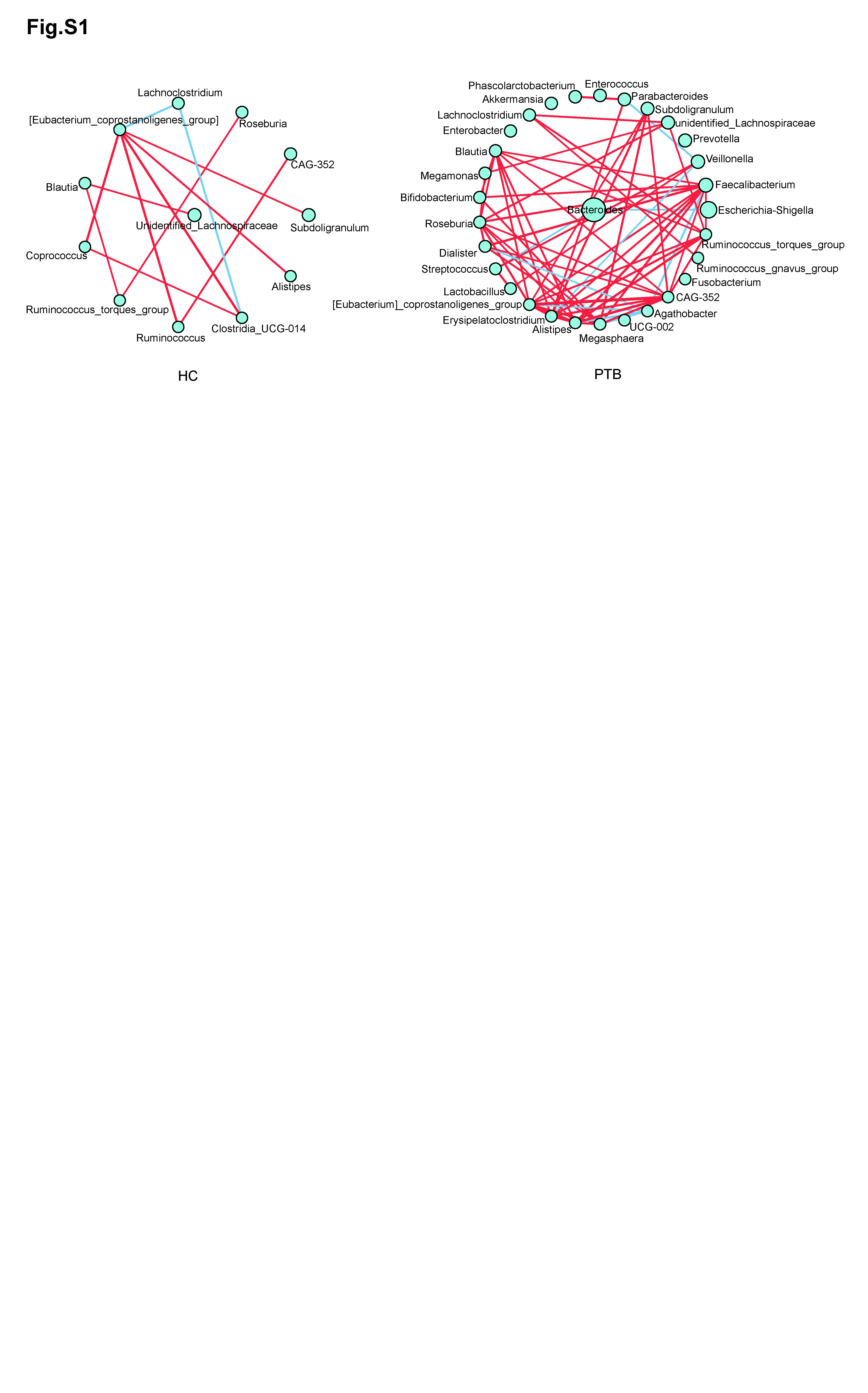

Supplement: Supplementary Figure 1 — The interaction network of intestinal flora in HCs and PTB patients. The interaction network of intestinal flora in HCs showed a simple relationship between the community members, and the interaction network of intestinal flora in PTBs had more positive and negative correlations among the bacteria. The correlation coefficients were calculated with the Sparse Correlations for Compositional (SparCC) data algorithm. Cytoscape version 3.4.0 was used for network construction. [file Image_1.jpeg]
