# Supplementary material for: The correlation between dysfunctional intestinal flora and pathology feature of patients with pulmonary tuberculosis
Source: Front Cell Infect Microbiol. 2022 Dec 21;12:1090889. doi: 10.3389/fcimb.2022.1090889 (PMC9811264; doi:10.3389/fcimb.2022.1090889)
Supplement: Supplementary file 2 [file Table_1.docx]

**Supplemmentary Table 1 Genera used to establish a random foreast classifier for the diagnosis of PTB**

| **Genus** | **Mean(HC)** | **Mean(PTB)** | **Enrich** |
| --- | --- | --- | --- |
| [Eubacterium]_ventriosum_group | 0.002365 | 0.001093 | HC |
| Monoglobus | 0.003390 | 0.002719 | HC |
| Dorea | 0.002973 | 0.001838 | HC |
| Roseburia | 0.016996 | 0.015328 | HC |
| Faecalibacterium | 0.147994 | 0.053117 | HC |
| Lactobacillus | 0.000626 | 0.010277 | PTB |
| Lachnospiraceae_NK4A136_group | 0.007189 | 0.003438 | HC |
| Incertae_Sedis | 0.002523 | 0.001710 | HC |
| Unidentified_Oscillospirales | 0.000656 | 0.000547 | HC |
| Fusicatenibacter | 0.001437 | 0.000387 | HC |
| [Eubacterium]_eligens_group | 0.004935 | 0.000965 | HC |
| Romboutsia | 0.007137 | 0.000645 | HC |
| Lachnospira | 0.004320 | 0.003244 | HC |
| Agathobacter | 0.033241 | 0.008571 | HC |
| Unidentified_Comamonadaceae | 0.000002 | 0.000489 | PTB |
| Unidentified_Ruminococcaceae | 0.005000 | 0.002656 | HC |
| [Clostridium]_innocuum_group | 0.000866 | 0.003074 | PTB |
| Pyramidobacter | 0.000072 | 0.000726 | PTB |
| Megasphaera | 0.010346 | 0.008256 | HC |
| Unidentified_Erysipelatoclostridiaceae | 0.000043 | 0.000941 | PTB |
| Anaerotruncus | 0.000027 | 0.000843 | PTB |
| Anaerostipes | 0.004938 | 0.001458 | HC |
| Desulfovibrio | 0.000814 | 0.001705 | PTB |
| Erysipelotrichaceae_UCG-003 | 0.003812 | 0.002037 | HC |
| Bifidobacterium | 0.050241 | 0.016748 | HC |
| Alloprevotella | 0.001134 | 0.001156 | PTB |
| Allisonella | 0.000512 | 0.000258 | HC |
| Clostridia_UCG-014 | 0.006960 | 0.004301 | HC |
| Sutterella | 0.000441 | 0.001322 | PTB |
| Methanobrevibacter | 0.000085 | 0.000729 | PTB |
